# Differential Gene Expression in Response to Salinity and Temperature in a *Haloarcula* Strain from Great Salt Lake, Utah

**DOI:** 10.3390/genes9010052

**Published:** 2018-01-22

**Authors:** Swati Almeida-Dalmet, Carol D. Litchfield, Patrick Gillevet, Bonnie K. Baxter

**Affiliations:** 1 Department of Environmental Science and Policy, George Mason University, 10900 University Blvd, Manassas, VA 20110, USA; swati_almeida2002@yahoo.com; 2 Department of Biology, George Mason University, 10900 University Blvd, Manassas, VA 20110, USA; pgilleve@gmu.edu; 3 Great Salt Lake Institute, Westminster College, 1840 South 1300 East, Salt Lake City, UT 84105, USA

**Keywords:** haloarchaea, salinity stress, temperature stress, Great Salt Lake, differential display, arbitrary primers, homeostasis

## Abstract

Haloarchaea that inhabit Great Salt Lake (GSL), a thalassohaline terminal lake, must respond to the fluctuating climate conditions of the elevated desert of Utah. We investigated how shifting environmental factors, specifically salinity and temperature, affected gene expression in the GSL haloarchaea, NA6-27, which we isolated from the hypersaline north arm of the lake. Combined data from cultivation, microscopy, lipid analysis, antibiotic sensitivity, and *16S rRNA* gene alignment, suggest that NA6-27 is a member of the *Haloarcula* genus. Our prior study demonstrated that archaea in the *Haloarcula* genus were stable in the GSL microbial community over seasons and years. In this study, RNA arbitrarily primed PCR (RAP-PCR) was used to determine the transcriptional responses of NA6-27 grown under suboptimal salinity and temperature conditions. We observed alteration of the expression of genes related to general stress responses, such as transcription, translation, replication, signal transduction, and energy metabolism. Of the ten genes that were expressed differentially under stress, eight of these genes responded in both conditions, highlighting this general response. We also noted gene regulation specific to salinity and temperature conditions, such as osmoregulation and transport. Taken together, these data indicate that the GSL *Haloarcula* strain, NA6-27, demonstrates both general and specific responses to salinity and/or temperature stress, and suggest a mechanistic model for homeostasis that may explain the stable presence of this genus in the community as environmental conditions shift.

## 1. Introduction

Great Salt Lake (GSL), Utah, is the largest lake in the western United States, and one of the largest thalassohaline terminal lakes in the world. The margins of GSL vary with precipitation and drought cycles, the meander line roughly measuring 122 km in length and 50 km in width, with an average depth of 4.3 m [1]. GSL was bisected by a railroad causeway in 1959 [2], isolating the hypersaline north arm of the lake, which currently has a salinity that varies between 270 and 300 g × L^−1^ (27–30%) total dissolved salts [3]. Variation in lake elevation directly impacts salinity. Thus, the microbial communities, composed largely of haloarchaea and bacteria [4] in this ecosystem, must respond to these changing conditions. Salinity gradients in the less saline south arm of the lake (140–150 g × L^−1^ total dissolved salts) have been shown to influence the composition of planktonic microbial communities [5,6]. However, a temporal study of the north arm microbiota shows communities that are more stable [4,7].

Great Salt Lake is situated in an elevated desert biome, and the average water temperature ranges from 0.5 °C in January to 26.7 °C in July [8], and up to 45 °C in the shallow margins [9]. An investigation of species present through the seasons probed the question of the impact of temperature as a driver for shifting communities, however, the microbial communities of this lake were, by and large, not affected by the change in temperature [4]. Since both salinity and temperature vary drastically in GSL, and since some species remain stable in the north arm, we hypothesized that some of the microorganisms respond to stress conditions by adjusting gene expression. 

Haloarchaea, like those in GSL, are ideal extremophiles for the study of genetics, as some can be easily cultivated and genetically manipulated [10]. Such models have improved our understanding of survival mechanisms of a microorganism under stress [11]. To maintain homeostasis under the challenging conditions of hypersalinity, haloarchaea must adapt. For example, some species may go into some sort of starvation-survival, long-term dormancy, as desiccation occurs [12]. Others may slow division but increase the genome copy number [13], perhaps for the purpose of phosphate storage [14]. However, if a particular species is a stable and dominant community member, despite fluctuations in environmental conditions, controlling homeostasis through differential gene expression would be a likely strategy.

Gene expression in haloarchaea has been well-studied, shedding light on the archaeal translational and transcriptional mechanisms, as well as small RNA structures and ribosome characterization [15,16,17,18]. Temperature and salinity have been shown to regulate the differential expression of genes in both *Halobacterium (Hbt.) salinarum* (sp. NRC-1) [19,20] and *Haloferax (Hfx.) volcanii* [21,22]. These studies indicated that genes for translation initiation factor, translation elongation factor, ABC transporter, protein kinase, cobalmin biosynthesis, glutamine amido transferase, and aspartate carbomyl transferase were commonly regulated under salt and temperature stresses. In addition, Coker and co-workers observed the regulation of genes for the potassium uptake system (*Trk*) and superoxide dismutase in *Hbt. salinarum* under salinity stress [19]. Even pigmentation of haloarchaea is impacted by salinity stress, as carotenoid biosynthesis changes occur with varying salinity in *Haloferax* and *Haloquadratum* species [23,24,25].

To test the hypothesis of differential gene expression as a mechanism for homeostasis, we selected NA6-27, which we show here is a member of the genus *Haloarcula.* Members of this genus were shown to be stable community members over seasons and years in our previous studies on GSL [7]. Would this haloarchaea strain from the dynamic GSL environment respond to environmental stress by adjusting gene expression? If so, which gene products would be impacted? Gene regulation has been previously observed in this genus with the haloarchaeon, *Haloarcula marismortui*. With respect to a temperature response, three RNA operons were shown to be differentially expressed at high and low temperatures [26]. Also, salinity changes caused a stress response in production of heat shock proteins, induction of carotenoid biosynthesis and sequestration of intracellular K^+^ and Cl^−^ [27]. Here, we report that the GSL strain, NA6-27, does indeed regulate gene expression in response to both salinity and temperature stress, suggesting a mechanism for its dominance over time in the lake’s microbial community.

## 2. Materials and Methods

### 2.1. Salt-Temperature Optima Study of NA6-27 

To determine optimum salinity and temperature for the GSL haloarchaeal isolate, NA6-27, the haloarchaeal standard growth medium [28] was supplemented with the following NaCl (w/v) concentrations: 3%, 8%, 10%, 15%, 20%, 25%, and 30%. For optimum temperatures at different salinities, the isolate was incubated at the following temperatures: 12, 24, 37, and 42 °C. We prevented water evaporation by including a beaker filled with water in the incubator. In addition, the plates were wrapped in parafilm.

Twenty-four well microtiter plates were used for salt-temperature study. Each plate contained three different concentrations of NaCl. For each concentration, the isolate was inoculated in triplicate. For each tested salinity, uninoculated “blank” wells of media corresponding to that salinity were used to normalize the background for the spectrophotometer readings. This procedure was repeated for temperature. The inoculum was grown in the haloarchaeal standard growth medium at 42 °C and 25% salinity to get 0.1 optical density (OD). In each plate, 1.5 mL of medium was inoculated with 110 µL of inoculum. A control plate with a known culture of *Har. marismortui* was used. Plates were read two times a week for 3 weeks. For plate readings, a Perkin-Elmer (Waltham, MA, USA) Spectracount Microplate Photometer was used with filter A_590_. The wells were monitored microscopically for the change in the size of cells as a response to osmotic or temperature stress. A Leica Microsystems (Wetzlar, Germany) DM LB2 phase contrast microscope was used to determine cell motility. 

### 2.2. Antibiotic Sensitivity, Biochemical Tests and Lipid Analysis

Growth on single carbon (5–50 mM) and nitrogen sources (2–20 mM) was determined under aerobic conditions by Phenotype Microarray Plates (Biolog, Hayward, CA, USA). Antibiotic containing discs or antibiotics in the appropriate liquid medium at concentration of 25–30 µg/mL were used to determine the antibiotic sensitivity of the isolate. A positive result was interpreted as a zone of inhibition of growth of 1 mm or more surrounding the disk. Phenotypic and biochemical tests were performed as described in the proposed minimal standards [29]. These tests included catalase and oxidase activity, indole formation, nitrate/nitrite reduction, hydrolysis of starch, gelatin and Tween 80. Anaerobic growth on arginine of the isolate was checked as described [30]. 

### 2.3. Thin Layer Chromatography 

The isolate was grown in a flask containing 100 mL of modified casamino acid medium [31] for 2 weeks at 37 °C on a shaker at 80× *g*. After growth, the culture was centrifuged at 6000× *g* for 15 min. The pellet was washed twice with sterile 25% solar salt water. For lipid analysis one dimensional thin layer chromatography (TLC) was used [32]. Lipids were extracted with chloroform/methanol (1:2 v/v) followed by chloroform/methanol (2:1 v/v). This latter extraction was performed three times, and water was used to facilitate the phase separation. All the extracts were pooled and dried in an automatic environmental SpeedVac Model 100 (Savant, New York, NY, USA). One dimensional TLC was used with a 19 channel 0.25 μL Whatman silica gel plates (Sigma Aldrich, San Luis, MO, USA), to which 20–40 µL of chloroform suspended extract was loaded in the appropriate lanes. Standard lipids for control included egg yolk, phosphatidic acid (PA), phosphatidyl choline (PC), phosphatidyl ethanol amine (PE), phosphatidyl glycerol (PG), and phosphatidyl glycerol phosphate (PGP). Control lipid extracts from known halophilic archaea included *Har. marismortui*, *Hbt. salinarum*, *Haloferax gibonsii*, and for known halophilic bacteria, included *Halomonas elogata*. For separation, a glass tank lined with a Whatman No. 1 filter paper saturated with the solvent was used. The chloroform/methanol/acetic acid/water (85:22.5:10:4 v/v) was used as the solvent system. After separation, plates were dried at room temperature for 30 min. Lipids were identified using reagents for the detection of specific groups: ninhydrin for tertiary amines, molybdenum blue for phospholipids, and orcinol for glycolipids. All plates were sprayed with H_2_SO_4_, and heated for the detection of total organic matter. Lipid analysis was done in triplicate to confirm the result.

### 2.4. 16S rRNA Gene Amplification

The colonies of haloarchaea NA6-27 were lysed in 50 µL of Tris-EDTA buffer pH 8.0 at 95 °C for 15 min, and then centrifuged at 2000× *g* for 5 min to pellet the cell debris. The DNA in the supernatant was directly used in amplification of *16S rRNA* gene with primers. The 1HKF (5′-ATTCCGGTTGATCCTGCCGG-3′) [33] and H1492R (5′-AAGTCGTAACAAGGTAAC-3′) [34] primers were used. Initial denaturation was at 94 °C for 4 min. Then, 40 cycles of denaturation at 94 °C for 1 min, annealing at 50 °C for 1 min, and extension at 72 °C for 3 min were done. Final extension was at 72 °C for 10 min. PCR products were cleaned and sequenced on an ABI 3130XL sequencer (Applied Biosystems, Foster City, CA, USA). The complete *16S rRNA* gene sequence was determined using primers covering around 1500 bp of the *16S rRNA* gene. The almost complete *16S rRNA* gene sequence was compared to sequences of members of *Halobacteriaceae* with the National Center for Biotechnology Information’s Basic Local Alignment Search Tool (BLAST) and the GenBank database [35].

### 2.5. Inoculum Preparation and Growth of NA6-27 at Different Conditions

To obtain the inoculum for gene expression study, NA6-27 was grown in a haloarchaeal standard growth medium [28] at 42 °C (optimum) in 20% w/v salinity until mid-exponential phase was reached. The cells were washed with 20% (w/v) salt water and resuspended to obtain 0.1 OD. Media were prepared at salt concentrations with 15%, 20%, and 27% (w/v) salinity, and incubated at 42 °C. Additionally, haloarchaeal medium with 20% (w/v) salt was incubated at 12, 37, and 42 °C. For each condition, three flasks each with 100 mL of haloarchaeal medium were inoculated with 1 mL of NA6-27 inoculum. The flasks were incubated at the stated temperatures until ~0.4–0.5 OD was reached. 

### 2.6. Arbitrary Primer Design

The 13-mer arbitrary primers were designed as described by [36] for *Har. marismortui* protein coding sequences [37]. A perl script was written for selection of high frequency 10-mer primers, which detected a gene only once, and also had either a start or stop codon to achieve preferential binding to either the 5′- or 3′-end of a gene [38]. This resulted in 43 different 10-mer primers, which together hit 3858 protein coding genes. Three extra bases were then added in front of each sequence to make a 13-mer primer to increase the reproducibility of the method [39]. Primers were purchased from Invitrogen (Carlsbad, CA, USA) and are listed in Table S1. Three high frequency primers (AP7, AP38, and AP39) with high G+C (77%) content, and detected the highest number of genes (>1000), were selected and used for reverse transcription (RT). The remaining 40 primers were used with these three primers in the differential display PCR.

### 2.7. RNA Extraction and Reverse Transcription PCR

RNA extraction involved 2.5 mL of phenol/chloroform (5% v/v) to 25 mL of the cell suspension, which was immediately transferred to −80 °C for 10 min to stabilize the mRNA. The cells were then harvested by centrifugation at 4 °C at 4000× *g* for 15 min. The cell pellet was stored at −80 °C until used for extraction. Total RNA was extracted using RNAbee (Tel-test; Friendswood, TX, USA) according to the manufacturer’s instructions. The RNA was treated with DNase, and extracted again with phenol [40]. Total RNA was visualized and quantified with a UV spectrophotometer.

Reverse transcription PCR (RT-PCR) was performed with three 13-mer arbitrary primers (Table S1) and Thermoscript Reverse Transcriptase (Invitrogen). Each reaction contained 10 μM of arbitrary primer, 1 μg of total RNA, 2 mM dNTPs, and distilled water to make total volume 10 μL. This mixture was incubated in the absence of buffer at 75 °C for 3 min in order to remove or denature the secondary structure of RNA, and then immediately placed on ice. Subsequently the following were added to each reaction: 5× complementary DNA (cDNA) synthesis buffer, 0.1 M DTT, RNaseOUT (40 units/μL), Thermosript RT (15 U/μL), 5 M betaine (Sigma Aldrich), and RNase free water to a total volume of 20 μL. An identical reaction without Thermoscript Reverse Transcriptase was performed to verify the absence of genomic DNA (−RT control). This mixture was briefly centrifuged, and subsequently incubated at 30 °C for 10 min, and then at 50 °C for 50 min. The reaction was terminated by heating at 85 °C for 5 min.

### 2.8. RNA Arbitrarily Primed PCR

The RNA Arbitrarily Primed PCR (RAP-PCR) was performed with a AmpliTaq Gold PCR kit (Applied Biosystems), and the reactions contained 1× buffer plus 2.5 mM MgCl_2_, and 5 units/µL Taq polymerase. This reaction mix was exposed to UV light (Stratagene UV stratalinker 2400) for 4 min to remove the contaminating DNA. Approximately 3 µL cDNA was added along with 2 mM dNTPs, 5 M betaine, and the primers at 10 µM; the first primer was the arbitrary primer which was used for the RT reaction, and the second primer was any other arbitrary primer (from the remaining 40 primers, see Table S1). The following conditions were used for PCR cycles: initial denaturation at 95 °C for 11 min, denaturation at 94 °C for 15 s, annealing at 40 °C for 2 min, extension at 72 °C for 1 min. Steps 2–4 were repeated for 40 cycles, and final extension of the PCR products was done at 72 °C for 10 min. The PCR products were separated on 2% agarose gel and analyzed to look for bands representing the differential expression of genes. To determine the expressed and repressed genes, the Kodak (Rochester, NY, USA) image analyzer software 1.0 was used to measure the intensity of each band. The differentially expressed band was selected and removed from the gel with a pipette tip and heated in diethylpyrocarbonate (DEPC) water at 95 °C for 2–3 min to elute the DNA from the gel. Negative control PCR reactions, with the UV-treated master mix and no added DNA, did not result in PCR products.

Appropriate primers were used to amplify the differentially expressed bands with the same methods as above. The selected PCR product was ligated into a TOPO 2.1 plasmid and cloned into *Escherichia coli* (strain TOP 10) cells according to the manufacturer’s instructions (TOPO TA cloning kit, Invitrogen). After cloning, the cells were spread on imMedia Amp Blue plates (Invitrogen) supplemented with 1% kanamycin (Sigma Aldrich) per plate, to avoid the growth of the satellite colonies.

For each differentially-expressed band, approximately 10–12 clones were randomly picked and grown in a 96 well microtiter plate containing, per well, 50 µL of Tris-EDTA buffer at pH 8.0. The colonies were heat-lysed at 95 °C for 15 min. The microtiter plate was then centrifuged for 8 min at 2000× *g* to precipitate the cell debris. The plasmid DNA in the supernatant was amplified with universal M13 primers (Invitrogen) with the Ampli Taq Gold PCR kit, as above, with the following conditions: 95 °C for 11 min, 95 °C for 30 s, annealing at 45 °C for 30 s, and 72 °C for 2 min with 5 s extension per cycle. After 40 cycles, the final extension was done at 72 °C for 10 min, and the products were kept at 4 °C. The PCR product was visualized on an agarose gel and purified using AMPure solution (Agencourt, Beverly, MA, USA). The Big Dye Terminator Kit (Applied Biosystems) was used with GeneAmp PCR system 9700 (Applied Biosystems) for the sequencing reaction. The product was purified using Sephadex G-50 gel filtration (Sigma Aldrich). The product was then dried and stored at −20 °C until it was reconstituted in Hi-Di Formamide to run on the SpectruMedix SCE 9610 (SpectruMedix LLC, Fullerton, CA, USA) capillary sequencer. The sequences were aligned using Sequencher software v4.1 (GeneCodes Corporation, Ann Arbor, MI, USA) and manually corrected when needed. The cloned sequences were analyzed by BLAST in GenBank [35]. The hit with the highest bit score was chosen as the closest gene function.

### 2.9. Confirmation of RNA Arbitrarily Primed PCR Fragments

To verify the differential expression of the fragments identified by RAP-PCR, the cDNA was synthesized as described above [22,41], with the same source of total RNA, arbitrary primer, and the Thermoscript RT-PCR system. Specific primers (Table S2) were designed with the online primer 3 plus tool [42], and were used at a final concentration of 10 µM. All of the primers had an annealing temperature of 60 °C. A confirmation PCR was then performed using specific primers for each sequence being verified. Cycles of PCR were as follows: 95 °C for 1 min, 60 °C for 1 min, 72 °C for 2 min for 25 cycles. For confirmation of differential gene expression, the correct size of the PCR product and the expression pattern was verified. In order to check the reproducibility, the experiments were performed in duplicate. 

### 2.10. Accession Numbers

The strain, NA6-27, was assigned the GenBank accession number JX306053 based on the *16S rRNA* gene sequence. In addition, the sequences of the ten identified gene fragments from this study have been deposited in GenBank under accession numbers from KT793928 to KT793937.

## 3. Results

### 3.1. Characterization of Optimal Growth Conditions for NA6-27 

Field work at the north arm of GSL produced a number of interesting haloarchaea cultivars [7], among them, the strain NA6-27, was isolated in February 2005 from the surface water at Rozel Point. Colonies of NA6-27, first observed growing at 37 °C on modified casamino acid medium [31] containing 25% NaCl, were circular, orange, and opaque with smooth consistency. These organisms were motile, and the cell shape triangular, consistent with known *Haloarcula* species [43].

**Figure 1:**
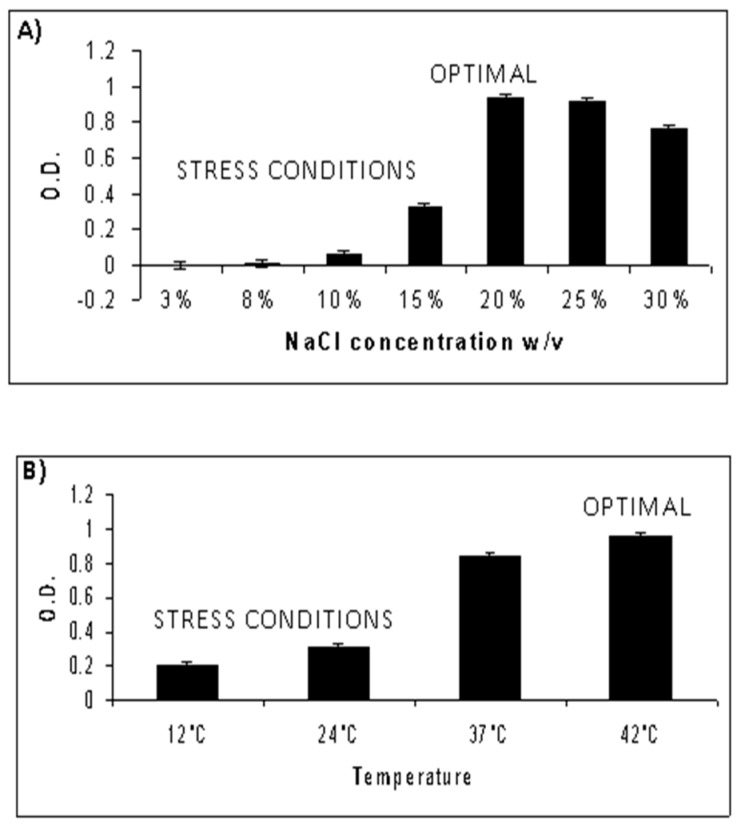
(**A**) Growth of the isolate NA6-27 in different salt concentrations at 37 °C after three weeks incubation in standard haloarchaeal medium. Error bars shows ± standard error. Stress conditions are indicated as is the optimal salinity; (**B**) Growth of isolate NA6-27 at different temperatures in 25% (w/v) salt after three weeks period. All cultures were grown in haloarchaeal standard medium. Error bars shows ± standard error. Stress conditions are indicated as is the optimal growth temperature. O.D: optical density.

Following isolation, we tested NA6-27 for optimal growth conditions. Cultivation over a range of salinity demonstrated an optimal salinity of 20% with the temperature held at 37 °C (Figure 1A). Growth was reduced only marginally as the salinity increased above the optimum, but growth was restricted significantly at the lower concentrations, 3 to 15%. When salinity was maintained at 20% and temperature was varied, NA6-27 grew best at 42 °C (Figure 1B), with slightly reduced growth measured at 37 °C. We tested growth at higher temperatures, 48 °C and 52 °C, and saw lower growth rates at all tested salinities (data not shown). In 20% NaCl media, we noticed a difference in lag phase under different temperatures; at 42 °C, the lag phase was less than an hour, whereas at 12 °C, the lag phase was between one and two days. We did not characterize stationary phase in these experiments. Since this strain did not grow well at the lower temperatures tested, 12 °C and 24 °C, we thus used 20% NaCl and 42 °C as “optimal” in the experiments described below. And the lower salinities and temperatures, at which growth was restricted, represent “stress” conditions for NA6-27.

### 3.2. Nutrient Utilization and Antibiotic Sensitivity for NA6-27

We employed Biolog plates, which test nutritional sources for microorganisms, to study the growth of the isolate on sole nitrogen (Table 1) and sole carbon (Table 2) sources. NA6-27 utilized many of the nitrogen sources, but it did not grow on sole amino acid nitrogen sources such as aspartic acid, aspargine, glutamic acid, and serine (Table 1). NA6-27 utilized half of the carbon sources tested (Table 2). Though glycerol phosphate as a carbon source was negative for growth, acetoacetic acid and pyruvic acid, which are by-products of glycerol metabolism, were utilized and gave a positive growth reaction.

To further identify this isolate, we performed biochemical tests and antibiotic sensitivity assays (data not shown). NA6-27 required 15–30% NaCl and 0.05 M MgCl_2_ for growth. The temperature range for growth was 10–48 °C, and the pH range was 7.0–8.0. It produced catalase and oxidase enzymes, grew anaerobically in nitrate, and reduced nitrate to nitrite with gas formation. It was not capable of hydrolysis of starch, gelatin, casein, or Tween 80. We treated NA6-27 with 10 different antibiotics. Archaea typically show sensitivity to antibiotics that affects their protein synthesis. The isolate was resistant to ampicillin, chloramphenicol, erythromycin, and penicillin, while showing sensitivity to anisomycin, bacitracin, neomycin, and novobiocin. These sensitivities are similar to *Har. marismortui* and *Har. vallismortis*, however, the latter is sensitive to erythromycin [44]. 

### 3.3. Lipid Analysis of NA6-27

Lipid characterization can aid in identification of haloarchaea at the genus level, since various types of lipids are characteristic of precise genera of the *Halobacteriaceae* [29]. When we treated NA6-27 with molybdenum blue, we observed the presence of several classes of phospholipids—PG, phosphatidylglycerosulfate (PGS), and PGP. Also, when sprayed with orcinol, the isolate showed the presence of glycolipids such as triglycosyldiether (TGD-2), which is the major glycolipid of *Haloarcula* [45], and an unidentified glycolipid, GL-1. These results provide evidence that this GSL isolate belongs the genus, *Haloarcula.*

### 3.4. Genetic Analysis of NA6-27 

Genomic DNA was extracted from a culture of NA6-27 and utilized as a PCR template to amplify the *16S rRNA* gene as described above, and the PCR product was sequenced. Our BLAST queries revealed the closest match for NA6-27 was *Har. quadrata str.* 801030/1, *Har. marismortui str.* ATCC 43049, and *Har. vallismortis str.* CGMCC1.2048 with 99% similarity [35]. Taken together, results based on colony and cell morphology, growth conditions, nutrient preferences, antibiotic sensitivity, and the lipid analyses all suggest similarity to other strains in the *Haloarcula* genus. The *16S rRNA* gene identity confirms this. Thus, we assume NA6-27 is a strain of the *Haloarcula* genus, the first such cultivated and described from GSL.

### 3.5. NA6-27 Gene Expression under Salinity and Temperature Stress

A transcriptional analysis of NA6-27, an environmental isolate, could tell us something about the stable members of the GSL microbial community and their response to environmental stress. In this case, we utilized a method particularly useful when complete genome sequence information is unavailable, RAP-PCR. Since archaeal mRNA do not possess poly-adenylated tails at the 3′-end, we cannot exploit oligo dT primers for cDNA amplification. Thus, random primers, usually hexamers or decamers, are typically used for RAP-PCR. This amplification method was used by Bidle with *Hfx. volcanii* [21], and Li and coworkers in *Shewanella* sp. WP3 [46] to show the impact of salinity on gene expression. The RAP-PCR assay is also useful to compare the genes expressed in two or more conditions simultaneously [47]. To confirm the differential expression of RAP-PCR fragments, a quantitative PCR method was used [22,41]. In our experiments, we are using 13-mer primers for cDNA amplification, which have been shown to enhance the cDNA amplification signal and also increase the reproducibility of the method [39] (Table S1).

In order to assess the effect of sub-optimal salinity and temperature on the gene expression of NA6-27, we designed RAP-PCR experiments to examine transcripts that could be analyzed later for their ascribed gene function. We attempted to mimic natural temperature and salinity variations to which haloarchaea in GSL would be exposed. For salinity stress experiments, NA6-27 cultures were grown at 15, 20, and 27% (w/v) NaCl, and incubated at the determined optimum temperature of 42 °C. Likewise, for observing temperature stress, the optimum salinity was held constant (20% w/v) and growth conditions varied in temperature: 12, 37, and 42 °C. 

For each culture grown under varying salinity or temperature, total RNA was extracted from NA6-27 (triplicate samples) and converted to cDNA as described above. The reverse transcription reactions were performed with primers having high G+C content (Table S1), since all sequenced haloarchaea, with one exception, have G+C rich genomes [48]. Another problem we addressed with respect to the likely G+C-rich genome, is that they are notorious for forming secondary structures within the mRNA [17]. This can cause premature termination of cDNA synthesis [49]. We observed that best RAP-PCR results were obtained with betaine, which destabilizes the secondary structures of mRNA and reduces mRNA melting temperature [49]. Because the usual temperature of 37 °C was unsuccessful, the RT reaction at a higher temperature (50 °C) was tried in the presence of betaine. This modification not only gave good products, but reduced undesired side products. 

**Figure 2:**
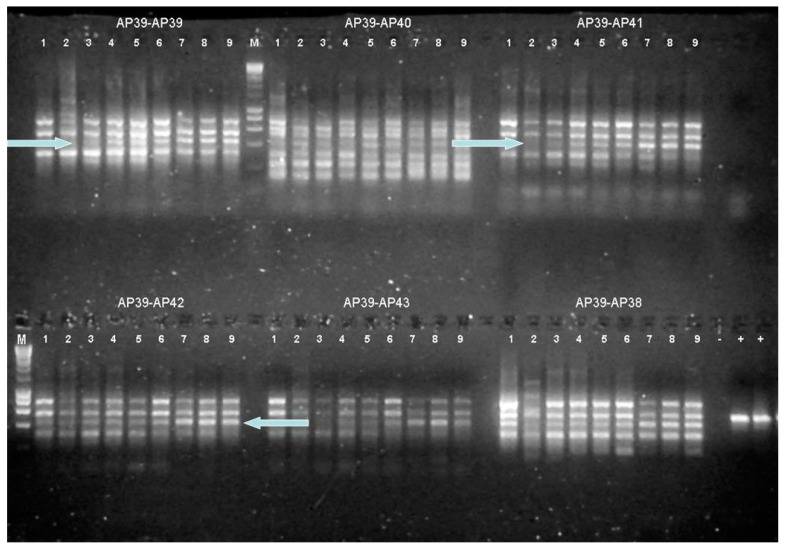
NA6-27 RNA arbitrarily primed PCR (RAP-PCR) fingerprint on a 2% agarose gel. Primers are indicated above the sample numbers. Arrows indicate the upregulated and downregulated genes. Each sample is in triplicate. 1, 2, 3 at 37 °C; 4, 5, 6 at 42 °C; and 7, 8, 9 at 12 °C (all at 20% salinity). M is the molecular weight standard, Gene Choice DNA Ladder 1 (Fisher Scientific, Hampton, NH, USA). The lower four bands from top to bottom represent 2000 bp, 1600 bp, 1000 bp, and 500 bp. +: Positive control (DNA of *Haloarcula marismortui*); −: Negative control (no DNA) are shown in lower right panel. Image shows only a portion of the data collected from reactions with different primer combinations.

The RT-produced cDNA provided a template for the RAP-PCR reactions, which utilized 120 different combinations of arbitrary 13-mer primers (Table S1), three from reverse transcription multiplied by 40 for differential display. The RAP-PCR products, which potentially revealed the differentially expressed genes, were reamplified, cloned, and sequenced. With each NA6-27 cDNA template, RAP-PCR produced 10–13 products of different size (Figure 2). After the identification of differential expression on agarose gels, the more intense (and thus more abundant) product bands that were verified in triplicate samples were selected. These were re-amplified, cloned, and sequenced for identification. The clone inserts ranged in size from 229–494 bp (Table 3).

### 3.6. Identification of Genes in Stress Experiments

**Figure 3:**
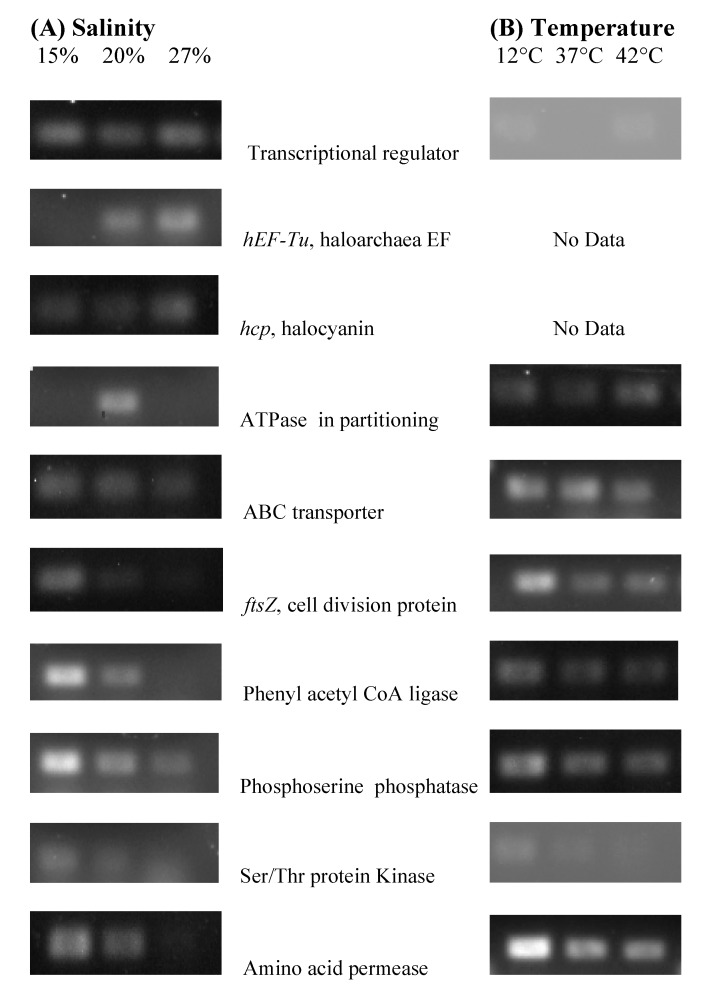
Confirmation of differential gene expression of RAP-PCR fragments in NA6-27 grown under the following conditions. (**A**) Salinity 15%, 20%, and 27% NaCl (w/v) and (**B**) Temperature 12, 37 and 42 °C. Quantitative PCR was performed using gene specific primers as described by Benson et al. [21,22,39]. Gel documentation photographs are aligned with data on genes for each growth condition.

Which genes were affected by salinity or temperature sub-optimal growth conditions? We used a BLAST query to align the clone sequences with candidates in GenBank. Out of the 45 total RAP-PCR products obtained from the total RNA of NA6-27, 12 matched with *16S* or *23S rRNA* genes. Those clones were not considered for further investigations, as they were artifacts often associated with differential display [50]. The analysis of the remaining products revealed ten candidate gene sequences that represented abundant transcripts in the stress experiments (Figure 3). A BLAST of these sequences gave us matches with previously sequenced haloarchaeal genes in GenBank with 85–100% similarity. Specific primers were designed based on the known sequence of candidate genes, and a confirmational PCR was done using these primers (Table S2). To note, *yrbE*, the gene for oxidoreductase, was observed in the clones, but could not be validated, since the primers used did not amplify the cDNA template. At 15% salinity, ABC transporter, *ftsZ,* transcription regulator, phenyl acetyl CoA ligase, phosphoserine phosphatase protein kinase, and amino acid permeases were significantly expressed (Figure 3), whereas at 27% salinity, *hEFTu* and halocyanin were highly expressed. At 12 °C, ABC transporter, *ftsZ* transcription regulator, phenyl acetyl CoA ligase, phosphoserine phosphatase, protein kinase, and amino acid permeases were expressed, whereas at 42 °C, ATPases involved in chromosome partitioning were more highly expressed. 

## 4. Discussion

The GSL is a terminal lake in a high desert ecosystem, and it is thus subjected to desiccation cycles and seasonal temperature fluctuation. Currently, the lake is experiencing a water crisis. Anthropomorphic impacts, such as upstream water demands [51] coupled with climate change [52] have impacted the lake elevation, as 2015 saw a historic low for the GSL north arm [53], and the levels continue to drop. Studies of stress impacts in the microbial foundation of the dynamic GSL ecosystem are paramount. To that end, we have isolated and characterized a dominant player in the GSL microbial community, NA6-27. This GSL strain is consistent with others in the *Haloarcula* genus in its morphology, biochemistry, and *16S rRNA* sequence, and it is a stable community member over time in the GSL north arm [4,7]. 

For the GSL *Haloarcula* strain, NA6-27, either low salinity or low temperature represent stress (Figure 1), but what are the cellular impacts of such stress on haloarchaea? In hypersaline environments, salinity decreases may be detrimental to microorganisms that are adapted to osmotically challenging brine. Cells must sense their osmotic environment and then compensate for the concentrations on the outside of the membrane. Haloarchaea produce and accumulate compatible solutes, which are small, organic molecules, such as sugars, amino acids or their derivatives, that can balance a cell osmotically without interfering with cellular processes [54,55,56,57]. This osmotic balancing is driven by many gene products that control biosynthesis, transport, and uptake. Temperature can affect solubility of salts in the brine, and can thus affect salinity, but the winter climate at GSL may cause additional impacts on the haloarchaea that live there. Decreases in temperature can slow microbial growth rates and enzyme activity in general, or may indirectly impact solubility and uptake of nutrients due to cell membrane modifications that reduce fluidity and transport rates [58]. With respect to both salinity and temperature, when conditions become sub-optimal, microorganisms may alter gene expression by either general or specific stress responses [59,60]. Indeed, we have observed changes in gene expression in global pathways like transcription, translation, and replication, but we have also observed expression changes specific to the lifestyle of this GSL *Haloarcula*, affecting gene products with roles in osmoregulation and in transport.

When *Har.* sp. NA6-27 was grown under sub-optimal conditions, some genes were differentially expressed under both salinity and temperature stress (Figure 2), which points to a generalized stress-induced system of gene regulation for the transcription and translation of particular sets of proteins.

Genes that are differentially regulated during the stress response may not have any obvious functional relevance to the tested condition [61], and may instead be more global. For example, we found that the expression of a transcriptional regulator was altered with both salinity and temperature shifts (Figure 3). Others have noted similar results with salinity [27] and during cold adaptation [62] in other haloarchaeal strains, indicating a link between environmental conditions and global transcription control. In addition to RNA synthesis, impacts on protein synthesis represent another typical response to a stress condition. Translation was impacted in our experiments with NA6-27; the *hEF-Tu* gene for halophilic elongation factor was repressed under salinity stress and upregulated as the salinity increased. The hEF-TU protein promotes the binding of the aminoacyl-transfer RNAs (tRNAs) to the ribosome during protein translation [63], and its repression under stress would slow growth. These results are consistent with general stress responses, which result from either salinity or temperature stress for NA6-27. 

Genes involved in replication and cell division should be upregulated under optimal conditions since the “optima” for NA6-27 were determined by growth rates of cultures. Indeed, the gene for *ATPase* involved in chromosome partitioning was expressed at the optimal salinity (20%) only. This gene was similar to gene on plasmid pNATPE01 of *Natrinema pellirubrum* DSM 15624. This gene is also present on the chromosome, and it is required for the plasmid replication during the cell division process [64]. In contrast, the gene for the *ftsZ* cell division protein was repressed at optimal conditions and highly expressed during stress, which correlates with prior studies on *Halobacterium* NRC-1 that demonstrated upregulation in cold temperatures [19]. The *ftsZ* gene encodes a GTP-binding tubulin-like cell division protein which is universal in bacteria and archaea [65] and controls cell division. Its regulation may suggest the increased need for intracellular organization at low temperatures [19]. Also, its gene product is an important substrate for *ftsH*, which acts as a molecular chaperone required to degrade partially unfolded and damaged proteins during temperature stress [66]. 

Genes involved in energy metabolism were also examined. As salinity increased in the optimal range, we observed upregulation of the *hcp* gene for halocyanin in NA6-27. Halocyanin is an electron transfer protein in the peripheral membrane of some bacteria and archaea [67,68], and is implicated in the electron transfer to the terminal oxidase in the respiration of halophilic archaea [67,69,70]. Nine such halocyanin-precursor-like genes have been identified in the *Har. marismortui* genome [71]. We did not observe a RAP-PCR product for these genes when varying the temperature conditions. 

Genes involved in signaling, for example protein kinases (*prk*) or proteins that regulate them, are linked to stress responses [72]. In our study, a protein kinase gene was repressed at both optimal salinity and temperature, but expressed during stress in NA6-27. Likewise, the phenyl acetyl CoA ligase gene was completely repressed at both the higher optimal salinity and temperature, but highly expressed under salinity stress. The phenyl acetyl CoA ligase is involved in the catabolism of phenyl acetic acid, and this enzyme forms phenyl acetyl CoA from phenyl acetate and CoA in the presence of ATP producing AMP [73]. These elevated levels of AMP may activate protein kinases. The genes for protein kinases are regulated in other haloarchaea under salt stress [19]. The function of protein kinase may be energy conservation, as Corton and others suggested; PRK stimulates a response regulator, which in turn switches off the biosynthetic pathways to preserve ATPs for more essential functions [72]. In addition, this may conserve the production of ion gradients, which would impact osmoregulation.

Altered expression of the genes for osmosensing and osmoregulation would be anticipated as a specific response for haloarchaea, and one might predict high expression levels of these genes would be correlated with high (optimal) salinity, but these systems are multifaceted. High salinity has been reported to stimulate intracellular accumulation of amino acids in haloarchaea [74], by affecting gene regulation of uptake and transporter proteins or amino acid biosynthesis pathways. However, we noted four genes related to osmophily were repressed or downregulated in the high salinity optimal conditions: (1) the gene for amino acid permease that allows amino acid uptake; (2) the phosphoserine phosphatase gene, which is involved in the biosynthesis of serine and ion transport [75]; (3) the phenyl acetyl CoA ligase gene, which is involved in the catabolism of phenyl acetic acid [73]; and (4) an iron ABC (ATP binding cassette) transporter, which also plays an important role in substrate uptake [76]. Others have noted similar observations regarding genes involved in osmoregulation being upregulated in lower salinities and repressed in higher salinities. Mei and co-workers observed that *Natrinema* sp. J7-2 accumulated the highest amount of osmoprotectant, free amino acids, when cells were grown under salinity stress [77]. Coker and others theorized that downregulation of an iron ATP transporter in a *Haloarcula* species at high (optimal) salinity, which would reduce uptake, could prevent the accumulation of toxic metal ions when the cells are actively growing [19]. These observations, and our experiments with NA6-27, may underscore the significance of the osmosensing function, which could be more important for haloarchaea under salt stress, and thus upregulated in lower salinities. The cells may need more acidic amino acids to stabilize their halophilic proteins and other structures, and to maintain activity at lower salinities. This response should be further examined in the context of other genes involved in osmophily.

In colder temperatures, such as winter conditions at GSL, NA6-27 would have lower rates of enzyme activity, membrane lipid fluidity, and diffusion across the membrane. Haloarchaea would, therefore, need more substrate to compensate [62]. It stands to reason that NA6-27 would upregulate transporter genes such as iron ABC transporter, phosphoserine phosphatase, and amino acid permeases under cold temperature stress at 12 °C. (Figure 2). Previous work noted that during cold adaptation, *Halohasta litchfieldiae* and *Halorubrum lacusprofundi* increased abundances of lipoproteins that target nutrients such as carbohydrates, branched chain amino acids, and various metal ions, especially iron [62]. Similar to low salinity, at low temperature, the amount of dissolved oxygen increases in the brine. To relieve the oxidative stress, more iron is needed to make iron superoxide dismutase [78].

## 5. Conclusions

We conclude that NA6-27, a member of the genus *Haloarcula*, which remains stable year-round in the north arm [8], adapts at the cellular level to the changing environmental conditions. In the work presented here, we observed an overlap in the gene expression responses to both salinity and temperature stress, which agrees with other studies [79,80]. This GSL *Haloarcula* strain reacts to environmental stress, in part, with general cellular responses. Global adjustments could certainly favor conserved energy when stressed, and continued growth under optimal conditions, both of which would lead to the dominance of the strain in the community. In order to fine-tune the response, NA6-27 also alters specific expression related to salinity, such as osmoregulation genes, or to temperature, such as transporter genes. Our study suggests a mechanistic model for homeostasis, and builds an understanding of the complexity of gene regulation in a single organism.

In the natural environment of GSL, this haloarchaeal species would be part of a larger community of microorganisms that could each be impacted by fluctuations in salinity and temperature, as well as interactions with metabolites from other members. Though we have examined NA6-27 in isolation, gene regulation in the context of the lake is likely to be more complex than seen in ocean studies [81]. One may see a gene expression pattern in a specific genus, or across taxa in particular categories of genes. Studies such as this one, on individual strains, will contribute to the story. However, projects that address gene expression in diverse halophilic microbial communities, under native fluctuating environmental conditions, are the obvious direction to take in understanding life at in a dynamic hypersaline environment.

## Figures and Tables

**Table 1:** Biolog assays. Nitrogen source utilization by Great Salt Lake (GSL) strain, NA6-27. Sources that resulted in observed growth are indicated as positive and are shown in the first two columns. Negative results are shown in the third and fourth columns.

Nitrogen Source Positive for NA6-27 Growth		Nitrogen Source Negative for NA6-27 Growth	
biuret	l-methionine	ammonia	adenosine
l-alanine	l-phenylalanine	l-aspargine	uric acid
l-arginine	l-valine	l-aspartic acid	Ala-gln
l-proline	d-asparagine	l-cysteine	Ala-glu
Gly-met	d-lysine	l-glutamic acid	Ala-thr
nitrate	d-valine	l-threonine	Met-ala
urea	ethanolamine	l-putrescine	l-valine
l-glutamine	uracil	agmatine	acetamide
glycine	Gly-asn	*N*-acetyl-d-glucosamine	guanosine
l-histidine	Gly-gln	*N*-acetyl-d-galactosamine	thymine
l-isoleucine	d-aspartic acid	uridine	xanthosine
l-leucine	Ala-gly	inosine	allontoin
l-lysine	Gly-glu	nitrite	amino-*n*-valeric ac
	Gly-met	l-serine	l-tryptophan
		l-ornithine	l-tyrosine
		ethelenediamine	l-citrulline
		formamide	xanthine
		d-galactosamine	l-citrulline
		d-mannosamine	

**Table 2:** Biolog assays. Carbon source utilization by GSL strain, NA6-27. Sources that resulted in observed growth are indicated in the left column, and sources that did not promote growth are listed in the right column.

Carbon Source Positive for NA6-27 Growth	Carbon Source Negative for NA6-27 Growth
d-glucose	2-amino ethanol
d-fructose	inosine
d-galactose	ketobutyric acid
d-mannose	maltose
acetoacetic acid	glyoxylic acid
pyruvic acid	uridine
glucuronamide	sucrose
l-malic acid	mallotriose
d-lactose	d,l-glycerol phosphate

**Table 3:** GenBank gene sequence matches for RNA arbitrarily primed-PCR products.

Primer Pairs	Product Size (bp)	Gene Match	Accession number	From Species	Alignment
39, 1	352	*hEF-Tu*, halophilic archaea elongation factor	KT793932	*Halobacterium marismortui*	100%
39, 3	434	Amino acid permease associated region	KT793929	*Haloarcula hispanica* ATCC 33960 chromosome II	99%
39, 6	229	Halocyanin precursor like	KT793937	*Haloarcula marismortui* ATCC 43049 chromosome I	86%
39, 28	330	ATPase involved in chromosome partitioning	KT793930	*Natrinema pellirubrum* DSM 15624 plasmid pNATPE01	98%
7, 30	494	*ftsZ*, cell division protein	KT793931	*Haloarcula japonica ftsZ2* gene for cell division protein *ftsZ2*	99%
7, 12	233	Transcriptional regulator	KT793936	*Haloarcula* sp. CBA1115	100%
38, 35	283	Phenyl acetyl coenzyme A ligase	KT793933	*Haloarcula* sp. CBA1115/*Haloarcula hispanica* N601 chromosome 2	99%
39, 38	342	Phosphoserine phosphotase	KT793934	*Haloarcula* sp. CBA1115	99%
39, 10	433	Iron ABC transporter ATP binding protein	KT793928	*Haloarcula* sp. CBA1115	99%
39, 38	430	Oxidoreductase aldo/keto reductase family	No accession number; PCR not comfirmedafter first trial	*Haloarcula marismortui* ATCC 43049 chromosome I	90%
39, 37	320	Serine/threonine protein kinase	KT793935	*Haloarcula* sp. CBA1115	100%

## Supplementary Materials

The following are available online at http://www.mdpi.com/2073-4425/9/1/52/s1: Table S1, List of 13-mer Primers, and Table S2, List of specific primers used in Real Time PCR.
