# Correction: Almeida-Dalmet, S.; et al. Differential Gene Expression in Response to Salinity and Temperature in a *Haloarcula* Strain from Great Salt Lake, Utah. *Genes* 2017, *9*, 52

**DOI:** 10.3390/genes9030146

**Published:** 2018-03-07

**Authors:** Swati Almeida-Dalmet, Carol D. Litchfield, Patrick Gillevet, Bonnie K. Baxter

**Affiliations:** 1 Department of Environmental Science and Policy, George Mason University, 10900 University Blvd, Manassas, VA 20110, USA; swati_almeida2002@yahoo.com; 2 Department of Biology, George Mason University, 10900 University Blvd, Manassas, VA 20110, USA; pgilleve@gmu.edu; 3 Great Salt Lake Institute, Westminster College, 1840 South 1300 East, Salt Lake City, UT 84105, USA

The authors wish to make the following changes to their paper [1]. In the original published version of Figure 2 (“Confirmation of differential gene expression …”), many of the photographic panels were missing. This figure has been replaced with a complete version (Figure 1). In addition, the position and numbering of Figures 2 and 3 have been exchanged to reflect the order in which they are mentioned in the text, and the references to these figures have been updated in the text.

We apologize for any inconvenience caused to the readers by this change. The manuscript will be updated and the original will remain online on the article webpage.

## Figures and Tables

**Figure 1:**
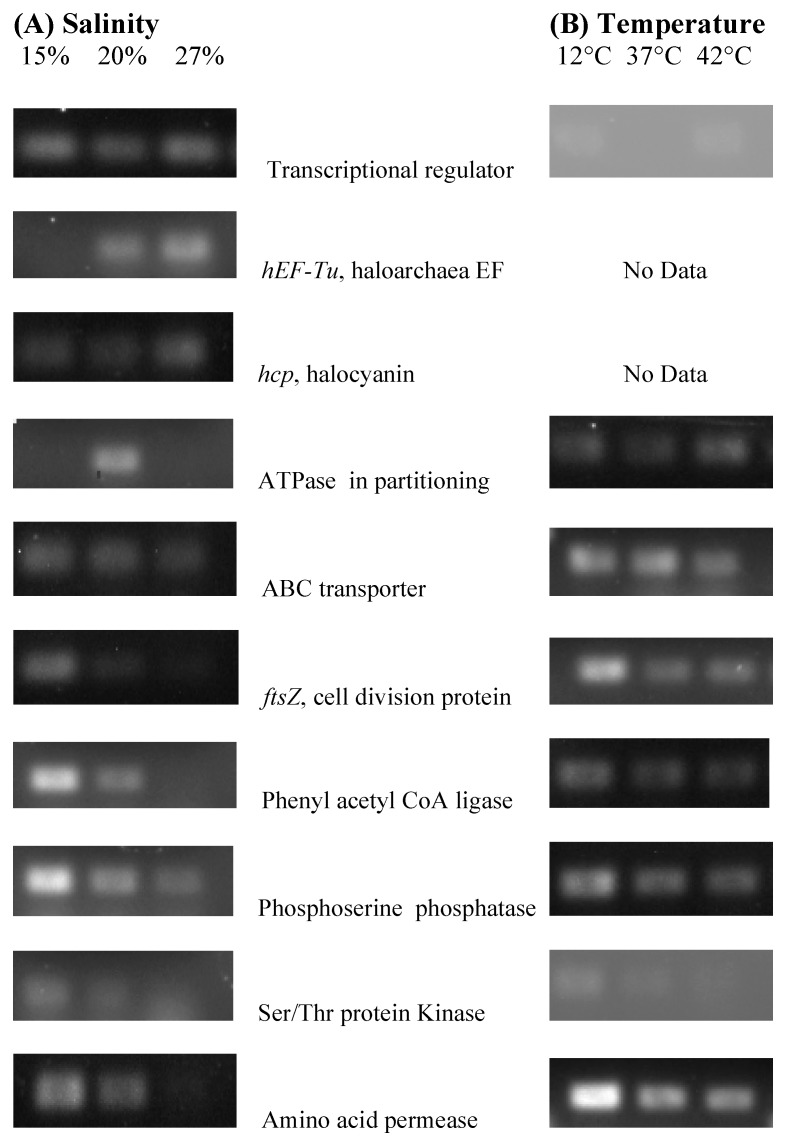
Confirmation of differential gene expression of RAP-PCR fragments in NA6-27 grown under the following conditions. (**A**) Salinity 15, 20, and 27% NaCl (w/v) and (**B**) Temperature 12, 37 and 42 °C. Quantitative PCR was performed using gene specific primers as described by Benson et al. [2,3,4]. Gel documentation photographs are aligned with data on genes for each growth condition.
