# Supplementary material for: Differential Gene Expression in Response to Salinity and Temperature in a Haloarcula Strain from Great Salt Lake, Utah
Source: Genes (Basel). 2018 Jan 22;9(1):52. doi: 10.3390/genes9010052 (PMC5793203; doi:10.3390/genes9010052)
Supplement: Supplementary file 1 [file genes-09-00052-s001.zip › Submitted zipped supplementary/Supplementary Table 2.doc]

**Supplementary Table 2.** List of specific primers used in Real Time PCR

| Differentially expressed Genes | Primer sequence |
| --- | --- |
| Transcriptional regulator gene | F: 5’CTGGTCAACAGCGACTTCAT-3’  R:3’TGACCAGTTTGGTCTTCTCG-5’ |
| hEF-Tu gene for halophilic elongation factor gene | F: 5’AAATCGTAGGCGTCAGTGCT-3’  R: 3’TCGAGTTCGCCTACGTCAT-5’ |
| Halocyanin precursor like gene | F:5’AGGTAGGTCCCAGCTTCCTC-3’  R: 3’CCGCATAACATCGTCTCAAA-5’ |
| Oxidoreductase | F: 5’GACGGGGTCATAGAAAGGAA-3’  R: 3’GGTAATCTGTGAGGGCTTCG-5’ |
| ATPase involved in chromosome partitioning gene | F: 5’CGATGATAACGATCCGTCAA-3’  R: 3’CTGAAAGCGTTGTGTTGTCC-5’ |
| Iron ABC transporter ATP-binding protein gene | F: 5’TCCTCCAGGAGAAGATGGAG-3’  R:3’TGGAGGACTTCGTTTTGCTT-5’ |
| fts Z gene for cell division protein gene | F: 5’GAGCCTCATCAACCTCGACT-3’  R:3’ CCTCCTCGGTCTTGTTCTTG-5’ |
| Phosphoserine phosphatase gene | F: 5’CGTCCGATCTCCGCTTTAT-3’  R: 3’GCGCTCAGCAGTGAGTGAAT-5’ |
| Phenyl acetyl coenzyme A ligase gene | F:5’ACGGCGAGGTAGAGTGCATA-3’  R: 3’GAACTGAGGAGCTGGGGG-5’ |
| Serine/Threonine protein kinase gene | F:5’GGGGCGTTCTGTAACGCCAA-3’  R: 3’TGTTCTTGACTGGCATGGAG-5’ |
| Amino acid permease gene | F: 5’GAGGTCGGGGACTACATTCA-3’  R: 3’GAAGTTGATACGCGAGGAC-5’ |
