# Supplementary material for: Differential Gene Expression in Response to Salinity and Temperature in a Haloarcula Strain from Great Salt Lake, Utah
Source: Genes (Basel). 2018 Jan 22;9(1):52. doi: 10.3390/genes9010052 (PMC5793203; doi:10.3390/genes9010052)
Supplement: Supplementary file 1 [file genes-09-00052-s001.zip › Submitted zipped supplementary/Supplementary Table1.doc]

Supplementary Table 1 . List of 13-mer Primers

| Primer Name | Sequence 5’-3’ | G+C content | No. of hits for genes |
| --- | --- | --- | --- |
| AP1 | CGACGGTCATCGA | 62 | 1244 |
| AP2 | CGACGGGTCATCG | 69 | 1231 |
| AP3 | CGACGGGACGATG | 69 | 1072 |
| AP4 | CGACGGCGTCATC | 69 | 1050 |
| AP5 | CGACGGTCACCGA | 57.1 | 1041 |
| AP6 | CGACGGCTCATCG | 57.1 | 1014 |
| **AP7^*^** | **CGACGGGTCACCG** | **77** | **1003** |
| AP8 | CGACGGGGCGATG | 71.4 | 951 |
| AP9 | CGACGGCGATGAC | 57.1 | 881 |
| AP10 | CGACGGCTCGATG | 57.1 | 848 |
| AP11 | CGACGGTCATCGG | 57.1 | 803 |
| AP12 | CGACGGCGTCACC | 71.4 | 788 |
| AP13 | CGACGGCGATGTC | 57.1 | 762 |
| AP14 | CGACGGTCAACGA | 42.8 | 761 |
| AP15 | CGACGGGCCGATG | 71.2 | 760 |
| AP16 | CGACGGTCATCGC | 57.1 | 738 |
| AP17 | CGACGGTCACCGG | 71.4 | 728 |
| AP18 | CGACGGGTCAACG | 57.1 | 700 |
| AP19 | CGACGGCGAGATG | 57.1 | 670 |
| AP20 | CGACGGTCGTCAC | 57.1 | 658 |
| AP21 | CGACGGGAGATGG | 57.1 | 627 |
| AP22 | CGACGGGGTCATC | 57.1 | 626 |
| AP23 | CGACGGGGCGTCA | 71.4 | 592 |
| AP24 | CGACGGCGTCAAC | 57.1 | 589 |
| AP25 | CGACGGCCTCATC | 57.1 | 574 |
| AP26 | CGACGGTCATCGT | 42.8 | 555 |
| AP27 | CGACGGGATGCGG | 71.4 | 545 |
| AP28 | CGACGGTCGTCAT | 42.8 | 523 |
| AP29 | CGACGGTTCATCG | 42.8 | 521 |
| AP30 | CGACGGGACCTCA | 57.1 | 515 |
| AP31 | CGACGGGGAGATG | 57.1 | 512 |
| AP32 | CGACGGGAACTCA | 57.1 | 509 |
| AP33 | CGACGGTCACCGT | 57.1 | 478 |
| AP34 | CGACGGATGACGA | 42.8 | 472 |
| AP35 | CGACGGGCGTCAC | 71.4 | 421 |
| AP36 | CGACGGGCGTCAT | 57.1 | 350 |
| AP37 | CGACGGATGACGG | 69 | 339 |
| **AP38 ^*^** | **CGACGGCGACGAC** | **77** | **3747** |
| **AP39 ^*^** | **CGACGGGACGACG** | **77** | **3262** |
| AP40 | CGACGGTCGACGA | 69 | 2876 |
| AP41 | CGACGGCGACGGC | 85 | 2792 |
| AP42 | CGACGGCGACGAG | 77 | 2784 |
| AP43 | CGACGGCGGCGAC | 85 | 2650 |

**^* primers with bold letters used for cDNA synthesis^**
